# Comparison of Beliefs about Health in Migrants and Swedish-Born Persons with Type 2 Diabetes

**DOI:** 10.3390/ijerph191912699

**Published:** 2022-10-04

**Authors:** Emina Hadziabdic, Katarina Hjelm

**Affiliations:** 1 Department of Health and Caring Sciences, Faculty of Health and Life Sciences, Linnaeus University, 351 95 Växjö, Sweden; 2 Department of Public Health and Caring Sciences, Uppsala University, 751 22 Uppsala, Sweden

**Keywords:** beliefs about health, diabetes mellitus, migrants

## Abstract

Background: Based on findings from previous qualitative studies comparing migrants and Swedish-born persons with diabetes mellitus type 2, it was hypothesized that dissimilarities exist in beliefs about health, including factors of importance for health between groups. Methods: A survey in a diabetes clinic in a migrant-dense area in Sweden. Results: Migrants generally perceived their health as poorer than Swedes, although it was not significantly different. Health mainly meant feeling well, being alert, and healthy and learning to live with disease despite of person’s origin. Studying factors of importance for health, migrants perceived knowledge about the body and treatment to influence health to a lower extent (*p* 0.009) and use of nature cure remedies to a higher extent (*p* 0.029) than Swedish-born persons. Conclusions: The findings partly supported the hypothesis that dissimilarities in factors of importance for health exist between migrants and Swedish-born persons, and need to be assessed.

## 1. Introduction

Today, approximately 281 million persons globally are migrants and the number is expected to increase in the future [1]. Many of these migrants have chronic illnesses such as diabetes mellitus [2,3] and this disease, mainly type 2, constitutes a growing worldwide public health problem [4]. The increasing prevalence of type 2 diabetes among migrants, persons who have moved to another country voluntarily as migrants or who have been compelled to move as refugees is well documented and leads to a grave public health task and a challenge for many European countries [5,6]. Language and cultural distance may influence health, self-care practices, education, and the type of healthcare required and provided [7], and make a significant impact on inequalities in diabetes care between persons of minority ethnic origin and the population of origin [5]. Health-related behavior, and thus health, is guided by individual beliefs about health and illness [8,9,10,11,12] that are culturally determined [13]. Therefore, it is important to compare beliefs about health, including factors that influence health, in migrants and the population of origin (in this study: foreign- and Swedish-born persons), diagnosed with type 2 diabetes, in order to meet healthcare needs related to the disease.

In previous qualitative studies, dissimilarities in beliefs about health have been shown in migrants of different foreign origin compared to Swedish-born persons diagnosed with diabetes mellitus [9,10,12,14].Although health was described similarly, as freedom from disease, ideas about factors contributing to health differed. Swedish-born persons described a healthy/regular lifestyle, being physically active, and eating a high-fiber diet. Persons from the former Yugoslavia emphasized enjoyment of life by deviating from the recommended diet and maintaining previous dietary habits [9,12,14]. Middle Eastern-born persons focused on feelings of mental well-being, stated adaptation to diabetes as important, and mentioned a number of “musts” concerning diet [9,14]. Use of different types of herbs as a complement to ordinary medication was frequently discussed among persons from Latin America [10], and persons from the former Yugoslavia described using herbal teas [9,12]. In general, Swedish-born persons discussed the influence of individual lifestyle and heredity while migrants focused on social factors (being a migrant, experiencing adaptational problems, limited finances, migrational experiences) and the importance of religion for health[9,12,14]. The studies indicated lower risk awareness and lower knowledge about the disease and its complications in migrants than in Swedish-born persons, later confirmed in a survey [15]. Dissimilarities in health beliefs may affect health-related behavior, including self-care [9,10,14]. Based on the findings from these previous qualitative hypothesis-generating studies [9,10,12,14] the next step in the research process is to test in a quantitative study the hypothesis that dissimilarities in beliefs about health, including factors of importance for health, exist between migrants and the population of origin diagnosed with type 2 diabetes, as no previous studies on this have been found. This new knowledge is important to verify findings from previous qualitative studies, as self-care and health-related behavior are determined and guided by the individual’s beliefs about health and illness. Healthcare staff should be aware of this in order to ensure patient autonomy and safety and to guarantee access to healthcare and good quality care for all patients.

### 1.1. Theoretical Framework

Health is a fundamental human right for all people, irrespective of socio-economic status, gender, religion, sexuality, nationality, or ethnic origin [16] and health can be defined as a state of well-being that is culturally defined, valued, and practiced, and reflected in individuals, enabling them to function in their daily lives[17]. Health is a multidimensional concept and is determined by different factors that are either impossible to influence, such as age, sex, and constitutional factors, and those that can be influenced, such as individual lifestyle, social and community networks, and general socio-economic, cultural and environmental conditions [18]. Health can be expressed from a subjective perspective, what is perceived by the individual, or self-rated health (SRH) [19]. Perceived health (or SRH) is a comprehensive and robust indicator for the state of general physical and mental health and is highly associated with various health outcomes that can be measured. Perceived health (or SRH) has been shown useful in identifying risk factors of poor health and a valid predictor of morbidity and mortality [19,20,21], for example, it is used in national population statistics by the Swedish Public Health Agency and by OECD in comparisons of European countries [22]. 

Health-related behavior, including self-care, and thus, health is determined by beliefs about health and illness held by the individual, grounded on the person’s knowledge and refined by experience. Individual beliefs are culturally determined, conveyed through language, and learned by socialization in contact with others in the family, other groups, and organizations in society [8,9,10,12,14]. Furthermore, beliefs about health and illness are in turn related to factors in the individual, nature, social relations, or the supernatural spheres [23]. Thus, the primary goal of healthcare is to provide culturally congruent care based on individual beliefs, practices, and values. In order to assess individual beliefs guiding health-related behavior, and thus health, healthcare needs to be designed in partnership between the individual and healthcare staff. Together, they can design an integrated form of care based on a combination of holistic, generic, and professional care knowledge, and in multi-professional collaboration, in order to achieve the goal of health or to prevent illness [17].

### 1.2. Aim

The aim of the study was to compare migrants and the population of origin (in this study: Swedish-born persons) diagnosed with type 2 diabetes, in regard to their beliefs about health, including perceived health and factors that influence health.

## 2. Materials and Methods

### 2.1. Design

A cross-sectional survey was implemented and a structured interview was used to collect data to describe and document beliefs about health, including perceived health, and factors that influence health in migrants and Swedish-born persons, and to facilitate studies of how these beliefs relate to the person’s background features [24].This study is part of a larger project focusing on migration, health, and diabetes with reporting data in different parts.

### 2.2. Procedure and Participants

Participants were recruited from a diabetes clinic in a healthcare center in an immigrant-dense area in a county in Sweden. All known migrants diagnosed with type 2 diabetes managed in the clinic were invited. Criteria for inclusion in the study were persons diagnosed with type 2 diabetes (ICD E 11) 25 aged ≥18 years and with a duration of diabetes ≥1 year. Participants with known psychiatric diagnoses (ICD F 00- F29/F60-F 99), registered in the medical journal, were excluded on the grounds that cognitive deficiency might influence the results.

The operation manager of the primary healthcare center was contacted by the researcher, to inform them about the study and to obtain permission for its implementation. After approval, the manager informed (orally and in written form) the diabetes specialist nurses about the study, and they then identified persons who met the inclusion criteria, based on digital records at the healthcare center. Written information about the study and requests to participate in the study were sent to identified migrants (*n*= 379) together with a prepaid reply envelope. The information was translated by authorized translators into the most common languages among migrants in Sweden [25], namely Arabic and Bosnian/Croatian/Serbian, and was included in the letter to persons of this origin. The investigators’ contact details were included in case the participants had any questions. Two reminders were sent three weeks apart to those who did not respond to the request. Fourteen envelopes were returned because they had the wrong postal address, and 52 persons replied that they did not want to participate in the study. A total of 242 persons did not answer. In total, 69 migrants returned the questionnaire, after which a similar procedure was performed to recruit 69 Swedish-born persons who were matched by gender and diagnosis of type 2 diabetes (ICD E 11) with the migrant group.

### 2.3. Data Collection

Data were collected, between September 2014 and March 2016, by structured interviews in a cross-sectional study based on questions developed in previous qualitative studies of individual beliefs about health [9,10,12,14].

The questions in the structured interview guide included three dimensions of beliefs about health: perceived health (or (SRH)) (one item); beliefs about health (four items) and factors influencing health (15 items). Perceived health was investigated with a single question—“How do you perceive your overall health status?”—which could be answered on an ordinal five-point scale with “very good”, “good” “fairly good” “bad” or “very bad”. In the data analysis, the responses “very good” and “good” were summarized, as were “bad” and “very bad”. The question has been well validated and offers a valuable summary of how patients perceive their overall health status (or SRH) 19. The patient’s own health perception (SRH) has been shown to predict future morbidity, use of health services, and mortality [19,22,26]. Participants responded to statements in the questionnaire concerning beliefs about health and factors influencing health by giving an answer on an ordinal 4-point scale: “yes”, “maybe”, “no” or “don’t know”. When analyzing data, the total number of responses “maybe” and “don’t know” was added up.

A registered nurse (bachelor’s and master’s degree exam) conducted the structured interviews in secluded venues at the primary healthcare center. When preferred by participants, the interviews were performed in the presence of a professional authorized interpreter (*n* = 40). The interpreter interpreted literally in first-person form, was neutral, and ensured confidentiality according to recommendations in Sweden[27]. A pilot study was held to test the interview guide for content and understanding and check the consistency in the interviews across subjects in ten foreign-born persons, and the interviews turned out well, with good quality, and thus, all material was included in the study.

### 2.4. Data Analysis

Descriptive statistics, in terms of frequencies and percentages, were used to study the three dimensions of health beliefs. Comparisons between groups, migrants and Swedish-born persons were made by tests of statistical significance by chi-squared test and Monte Carlo based *p*-values significant 2-sided, with *p* < 0.05 considered statistically significant. Furthermore, the influence of education, gender, and ethnic origin in terms of country of birth (born in Sweden, born in a European country, or born in a non-European country) was tested. In the next stage, binary regression analysis was carried out with calculation of the odds ratio (OR, 95% CI) to assess the statistical significance of the association between dependent variables in terms of three factors influencing health (influence of knowledge about body and treatment; finances/financial situation; use of nature cure remedies) and the independent demographical variables educational level (high (university) and low (no education/primary/secondary school)), gender (female and male) and ethnic origin in terms of country of birth (born in Sweden, born in a European country, or born in a non-European country). The analysis was performed with the help of SPSS version 26 (SPSS Inc, Chicago, IL, USA).

## 3. Results

The study included 138 participants, whereof 69 were migrants (37 men and 32 women) and 69 were born in Sweden (37 men and 32 women). Age varied from 33–90 years in the group of migrants and from 48–91 years in Swedish-born persons (see Table 1). The group of migrants originated mainly from the former Yugoslavia and from the Middle East (see Table 2).

### 3.1. Perceived Health

In general, migrants perceived their health as poorer than Swedish-born persons, although the results were not significant (45% vs. 60%, see Table 3).

### 3.2. Beliefs about Health

Beliefs about what health means did not differ between the studied groups. Health meant mainly feeling well and being alert and healthy (98 vs. 98%) (see Table 4); and learning to live with the disease (87% vs. 85%). About half of the respondents perceived health as being free from disease (50% vs. 54%) and most doubts were expressed concerning the influence of wealth (38% vs. 24%). There were no differences in relation to gender, education, and ethnic origin in terms of being born in Sweden, Europe, or outside Europe.

### 3.3. Factors Influencing Health

The results showed no differences in beliefs about health (see Table 5) concerning the influence of different factors important for health, with the exception of the influence of knowledge about the body and treatment and use of nature cure remedies. Migrants considered the influence of knowledge about the body and treatment to a lower extent than Swedish-born persons (72% vs. 87%, *p* = 0.009) and the use of nature cure remedies to a higher extent (52% vs. 28% *p* = 0.029). However, there was a tendency for Swedish-born persons to perceive finances/financial situation as influencing health to a higher extent than migrants (66% vs. 77%, *p* = 0.056).

When studying the influence of gender, education, and ethnic origin (being born in Sweden, in Europe, or outside Europe), significant differences were shown only concerning the influence of knowledge on health. Thus, being a woman, having low education, or being of European origin had a negative influence (data not shown; *p* 0.041, 0.045, 0.012). When adjusting for these factors in binary logistic regression analysis (Table 6), being a migrant and educational level were still related factors. Believing that knowledge about body and treatment was an important factor for health was associated with being born in a European country, and the use of nature cure remedies was also important. The influence of nature cure remedies was also related to being born in a non-European country, although with a slightly weaker association. The importance of the economy for health was related to high educational level.

However, the results showed that the majority of the respondents (Table 5; approximately 80–90%), irrespective of origin, perceived that individual lifestyle factors, such as inactivity, smoking, diet, stress, and social and emotional factors expressed as thoughts/emotions and support from others (family, friends), had the greatest influence on health. Most hesitation was found (approximately 30–50%) concerning the influence of whether the person was a believer, and celebrating feasts and traditions.

## 4. Discussion

This study is unique since there are no previous studies investigating data regarding beliefs about health including perceived health and factors that influence health by comparing migrants and the population of origin (Swedish-born persons) diagnosed with type 2 diabetes. The results of this study found that migrants in general perceived their health as poorer than Swedish-born persons did. The findings partly supported the hypothesis generated in qualitative studies concerning dissimilarities in beliefs about health [9,10,12,14]. The study showed that beliefs about what health means did not differ but that there were dissimilarities in beliefs about factors of importance for health. Migrants considered the influence of knowledge about the body and treatment as factors influencing health (individual factors [23]) to a lower extent than Swedish-born persons did, and believed in the use of nature cure remedies (natural factors [23]) to a higher extent. There was a tendency for Swedish-born persons to perceive finances/financial situation (social factor [23]) as influencing health to a higher extent than migrants. Beliefs about the influence of knowledge and treatment on health were also correlated to being a woman, having low education, and being of European origin. In this study, migrants, particularly women and those of European origin, had a lower educational level than other subjects, which might explain these results. Previously, migrants with type 2 diabetes have been shown to have lower knowledge about diabetes than Swedish-born persons [15] and more frequent use of nature cure remedies has been indicated in qualitative studies in some migrant groups [9,10,12]. Whether the use of nature cure remedies influences health in persons with diabetes has been studied only to a limited extent. However, as individual beliefs are based on the person’s knowledge and guide health-related behaviors, including self-care, it is important to assess them and to plan diabetes education based on individual beliefs about health [7].

The results showed that migrants perceived their health in general to be poorer than Swedish-born persons, findings that support previous results [3,28] showing migrants to report poorer health status than the populations of origin in northern Europe. The findings might be explained in relation to the heterogeneity of the migrant population connected to their cultural, ethnic, and religious backgrounds, their socio-economic status, and their education and migratory status (refugee or labor migrants), and those factors may be barriers to their access to healthcare [29], thereby leading to poor health. The migrant population in Sweden is a heterogeneous group, including people who came to Sweden as labor migrants when they were young and have grown old in Sweden (mainly from Finland), as well as those persons who have migrated as refugees (migrants mainly from the former Yugoslavia, the Middle East, Afghanistan, and North Africa) [30]. This is to be added to having a chronic disease such as diabetes, requiring adaptation and lifestyle changes that may also influence self-rated, or perceived, health negatively [31].Self-rated, or perceived, health is a powerful measure of clinical outcomes such as morbidity and mortality [20], and has been used in national population statistics (every second year, annually since 1986) by The Swedish Public Health Agency [26] to study how the population feels and to follow changes in health over time, and by the OECD to review health status in European countries [22]. Therefore, it is important to be aware of the results of this study when developing diabetes care for this population.

In this study, beliefs about health were expressed mainly as related to individual factors such as feeling well and being alert, healthy, and learning to live with the disease. Health is then defined as a state of well-being that is culturally defined, valued and practiced, and reflected in individuals, enabling them to function in their daily lives [17]. Although there were similarities in beliefs between the migrants and Swedish-born persons in this study as regards what health means, it is important in the assessment of the individual and individual beliefs to identify both similarities and diversities in order to be able to provide culturally congruent care, adapted as far as possible to the individuals’ preferred goal of health [17]. Providing integrated culturally congruent care, including lay beliefs and not only professional beliefs, and based on a holistic approach delivered by a multidisciplinary team, will not only benefit migrants but also will reduce the costs of public health in the host countries.

It is valuable knowing that dissimilarities were found in health beliefs concerning factors of importance for health in terms of knowledge about the body and treatment, and the use of nature remedies. Migrants considered the influence of knowledge about the body and treatment to a lower extent than Swedish-born persons, and the use of nature cure remedies to a higher extent, which confirmed the results from previous qualitative studies [9,10,12,14]. It was also evident that being a woman, having low education, and being of European origin (lower educational level than non-Europeans in the studied population) influenced factors of importance for health which indicated that limited knowledge influenced beliefs about health. Social determinants of health, such as gender, cultural background, and having a low educational level, can negatively affect the health of foreign-born persons and the quality of care they receive [31]. Therefore, it is important when planning care for this population to address social and cultural structural dimensions as determinants of health [18]in order to provide holistic, person-centered, and integrative care considering both lay and professional beliefs [17].

### Study Limitations

The heterogeneity of the studied migrant population might be seen as a limitation. However, the group mainly included persons born in countries in the Middle East and the former Yugoslavia, thus it represented the largest group of migrants in Sweden [25]. They had similar socio-economic backgrounds, and both genders were equally represented. Another possible limitation could be that data from a single institution (or area) has been studied. However, this was the area most densely populated by migrants in the city, so it is likely to have covered the variation of migrants representing the migrant population in Sweden. Furthermore, the rate of dropouts could have been influenced but analysis of them did not show any significant differences when matched to the registered population of migrants diagnosed with type 2 diabetes concerning gender, age, and country of birth.

During the time period since data were collected (September 2014 to March 2016) things might have changed. The focus of this study (being one part of a larger project) is on individual beliefs about health compared between migrants and a population of origin, not previously studied, and not on the temporal development of beliefs. Thus, unique basic knowledge from a cross-sectional study is added, which is a strength of the investigation. However, for both groups circumstances might have changed emphasizing the importance of knowledge about beliefs about health including factors of importance for health. This knowledge is even more important in light of changes in society and the ongoing global migration.

Regardless of these limitations, the study results partly support findings from previous qualitative studies [9,10,12,14] concerning beliefs about health and illness when comparing migrants and Swedish-born persons diagnosed with diabetes mellitus.

## 5. Conclusions

The findings of this study partly supported the hypothesis of dissimilarities in beliefs about health. Beliefs about what health means did not differ but dissimilarities in beliefs about factors of importance for health were shown to exist between migrants living in Sweden and Swedish-born persons diagnosed with type 2 diabetes. Concerning the influence of different factors important for health, migrants perceived that knowledge about the body and treatment influenced beliefs about health to a lower extent and they used nature cure remedies to a higher extent than Swedish-born persons. The present study contributes unique basic knowledge and highlights the importance of identifying individual beliefs about health and thereby improving self-care behavior in order to address patient autonomy and safety, and to promote health and prevent ill health and illness.

## Figures and Tables

**Table 1:** Characteristics of the study population.

Variable	Swedish-Born *n* (%)	Migrants *n* (%)
Gender		
Male	37 (54)	37 (54)
Female	32 (46)	32 (46)
Age		
18–64 years	35 (26)	44 (64)
≥65 years	44 (64)	25 (36)
Educational level		
No education	0 (0)	10 (15)
Primary school < 9 years	25 (36)	27 (39)
Secondary school ≤ 12 years	24 (35)	17 (25)
University ≤ 2 years	7 (10)	9 (13)
University > 2 years	13 (19)	6 (9)
Employment status		
Employed	20 (29)	14 (20)
Unemployed	2 (3)	12 (17)
Student	0 (0)	2 (3)
Sick leave	0 (0)	11 (16)
Early retirement	10 (14)	12 (17)
Retirement	37 (54)	18 (26)
Period of being diagnosed with diabetes in years		
<10 years	28 (40)	39 (58)
≥10 years	41 (60)	30 (42)
Mean (SD)	12 (7)	11 (8)
Diabetes treatment		
Diet	9 (13)	10 (15)
Oral medication	33 (48)	38 (55)
Insulin	16 (23)	8 (12)
Combination Oral medication and insulin	11 (16)	13 (19)
Glycemic control		
<52 (mmol/mol) good	26 (38)	35 (51)
52–70 (mmol/mol) acceptable	40 (58)	21 (30)
>70 (mmol/mol) poor	3 (4)	13 (19)
Mean (SD)	55 (10)	58 (15)
Reason of migration		
Refugee		50 (73)
Employment		7 (10)
Relative		12 (17)
Years of stay in Sweden, Mean (SD)		25 (17)

**Table 2:** Country of birth of the study population among migrants and Swedish-born persons with type 2 diabetes living in Sweden.

Country of Birth	*n*
Sweden	69
Former Yugoslavia Bosnia Kosovo Croatia	14 9 3 2
Turkey	8
Poland	4
Finland	4
Italy	1
Syria	14
Iraq	12
Chile	5
Lebanon	3
Sri Lanka	1
Burma	1
Burundi	1
Somalia	1
Total	138

**Table 3:** Perceived health among migrants and Swedish-born persons with type 2 diabetes living in Sweden.

		Migrants *n* (%)	Swedish-Born *n* (%)	Total *n* (%)	*p*-Value
Perceived health	Very good/good	30 (45)	40 (60)	70 (52)	0.109
	Fairly good	22 (33)	20 (30)	42 (31)	
	Bad/very bad	15 (22)	7 (10)	22 (16)	

**Table 4:** Beliefs about what health mean in migrants and Swedish-born persons with type 2 diabetes living in Sweden.

Beliefs about Health		Migrants *n* (%)	Swedish-Born *n* (%)	*n* (%)	*p*-Value
To be free from disease	Yes	25 (50)	31 (54)	56 (52)	0.895
	No	13 (26)	14 (25)	27 (25)	
	Maybe/don’t know	12 (24)	12 (21)	24 (22)	
To feel good and feel fit and healthy	Yes	49 (98)	55 (98)	104 (98)	0.995
	No	0	0	0	
	Maybe/don’t know	1(2)	1 (2)	2 (2)	
Wealth	Yes	24 (87)	32 (59)	56 (55)	0.270
	No	5 (11)	9 (17)	14 (14)	
	Maybe/don’t know	18 (38)	13 (24)	31 (31)	
Learning to live with the disease	Yes	41 (87)	47 (85)	88 (86)	0.954
	No	2 (4)	3 (6)	5 (5)	
	Maybe/don’t know	4 (8)	5 (9)	9 (9)	

**Table 5:** Beliefs about factors of importance for health among migrants and Swedish-born persons with type 2 diabetes living in Sweden.

Factors of Importance for Health		Migrants *n* (%)	Swedish-Born *n* (%)	Total *n* (%)	*p*-Value
Knowledge about body and treatment ^1^	Yes	42 (72)	54 (87)	96(80)	0.009
	No	8 (14)	0 (0)	8 (7)	
	Maybe/don’t know	8 (14)	8 (13)	16 (13)	
Finances/financial situation ^1^	Yes	35 (61)	48 (77)	83 (70)	0.056
	No	15 (26)	6 (10)	21 (18)	
	Maybe/don’t know	7 (12)	8 (13)	15 (13)	
Gainful employment or being occupied with something ^1^	Yes	47 (83)	56 (90)	103 (87)	0.372
	No	5 (9)	2 (3)	7 (6)	
	Maybe/don’t know	5 (8)	4 (7)	9 (8)	
Fulfilling different roles in life ^3^	Yes	44 (80)	44 (73)	88 (77)	0.674
	No	4 (7)	5 (8)	9 (8)	
	Maybe/don’t know	7 (13)	11 (18)	18 (16)	
Stress ^1^	Yes	53 (95)	58 (93)	111 (94)	0.942
	No	2 (4)	3 (5)	5 (4)	
	Maybe/don’t know	1 (2)	1 (2)	2 (2)	
Thoughts and feelings ^1^	Yes	49 (91)	53 (88)	102 (90)	0.912
	No	2 (4)	30(5)	5 (4)	
	Maybe/don’t know	3 (6)	4 (7)	7(6)	
Relations to other ^3^	Yes	41 (77)	50 (82)	91 (80)	0.586
	No	4 (8)	2 (3)	6 (5)	
	Maybe/don’t know	8 (15)	9 (15)	17 (15)	
Support from others (family and friends) ^3^	Yes	48 (90)	50 (82)	98 (85)	0.301
	No	4 (7)	4 (7)	8 (7)	
	Maybe/don’t know	2 (3)	7 (11)	9 (8)	
Heredity ^1^	Yes	45 (78)	55 (89)	100 (83)	0.261
	No	7 (12)	4 (6)	11 (9)	
	Maybe/don’t know	6 (10)	3 (5)	9 (7)	
Inactivity ^1^	Yes	52 (96)	54 (87)	106 (91)	0.190
	No	2 (4)	5 (8)	7 (6)	
	Maybe/don’t know	0 (0)	3 (5)	3 (3)	
Smoking	Yes	51 (94)	58 (95)	109 (94)	0.707
	No	2 (4)	1 (2)	3 (3)	
	Maybe/don’t know	1 (2)	2 (3)	3 (3)	
Influence of faith ^4^	Yes	23 (43)	20 (32)	49 (38)	0.546
	No	19 (36)	22 (36)	41 (36)	
	Maybe/don’t know	11 (21)	19 (31)	30 (26)	
Diet ^1^	Yes	49 (91)	58 (94)	107 (92)	0.366
	No	4 (7)	2 (3)	6 (5)	
	Maybe/don’t know	1 (2)	2 (3)	3 (3)	
Celebrating holidays and traditions ^4^	Yes	27(52)	18 (32)	45 (42)	0.064
	No	16 (31)	19 (34)	35 (33)	
	Maybe/don’t know	9 (17)	19 (34)	28 (26)	
Use of nature cure remedies ^2^	Yes	27(52)	17 (28)	44 (39)	0.029
	No	12 (23)	24 (39)	36 (32)	
	Maybe/don’t know	13 (25)	20 (33)	33 (29)	

According to the lay theory model of illness causation [23] these factors were categorized in relation to factors in the individual ^1^, the nature ^2^, the social ^3^ and supernatural world ^4^.

**Table 6:** Multifactorial influence on factors of importance for health among migrants and Swedish-born persons diagnosed with type 2 diabetes living in Sweden. Results from multiple logistic regression analysis with significant influence shown for independent variables.

Dependent Variable	Independent Variables	*p*-Value	Odds Ratio (95% CI)
Factors of importance for health:			
(1) Knowledge about body and treatment	Low educational level	0. 523	0. 7 (0.2–2.0)
	Gender (female)	0. 243	0. 6 (0.2–1. 4.6)
	Ethnic origin:		
	- born in a European country	0. 039	0. 3 (0.1–0.9)
	- born in a non-European country	0. 113	0. 4 (0.1–1.2)
(2) Finances/financial situation	High educational level	0. 013	4. 3 (1.4–13.7)
	Gender (female)	0. 740	0. 9 (0.3–1.9)
	Ethnic origin:		
	- born in a European country	0. 300	0. 6 (0.2–1.6)
	- born in a non-European country	0. 057	0. 3 (0.1–1.0)
(3) Use of nature cure remedies	Low educational level	0. 703	1. 1 (0.4–2.8)
	Gender (female)	0. 904	0. 9 (0.4–2.0)
	Ethnic origin:		
	- born in a European country	0. 038	2. 9 (1.0–8.0)
	- born in a non-European country	0. 040	2. 6 (1.0–6.5)

Multiple regression analysis was performed in SPSS. Co-variates (variables with *p* < 0.05 in bivariate analysis) was entered as categorical variables. Educational level was dichotomized as high (university) and low (no education/primary/secondary school) level of education. Gender was dichotomized as female and male. Ethnic origin was dichotomized as being a migrant (born in a European country or born in a non-European country) and born in Sweden.

## Data Availability

In order to protect the integrity, anonymity and confidentiality of the respondents, data will not be shared.
